# Adhesion Pulmonary Nodules Detection Based on Dot-Filter and Extracting Centerline Algorithm

**DOI:** 10.1155/2015/597313

**Published:** 2015-05-19

**Authors:** Liwei Liu, Xin Wang, Yang Li, Liping Wang, Jianghui Dong

**Affiliations:** ^1^College of Computer Science and Engineering, Changchun University of Technology, Changchun 130012, China; ^2^Sansom Institute for Health Research and School of Pharmacy and Medical Sciences, University of South Australia, Adelaide, SA 5001, Australia; ^3^School of Natural and Built Environments, University of South Australia, Adelaide, SA 5095, Australia

## Abstract

A suspected pulmonary nodule detection method was proposed based on dot-filter and extracting centerline algorithm. In this paper, we focus on the distinguishing adhesion pulmonary nodules attached to vessels in two-dimensional (2D) lung computed tomography (CT) images. Firstly, the dot-filter based on Hessian matrix was constructed to enhance the circular area of the pulmonary CT images, which enhanced the circular suspected pulmonary nodule and suppresses the line-like areas. Secondly, to detect the nondistinguishable attached pulmonary nodules by the dot-filter, an algorithm based on extracting centerline was developed to enhance the circle area formed by the end or head of the vessels including the intersection of the lines. 20 sets of CT images were used in the experiments. In addition, 20 true/false nodules extracted were used to test the function of classifier. The experimental results show that the method based on dot-filter and extracting centerline algorithm can detect the attached pulmonary nodules accurately, which is a basis for further studies on the pulmonary nodule detection and diagnose.

## 1. Introduction

Pulmonary nodules are small masses of tissue in the lung, are prevalent findings on chest and abdominal CT scans, and can be cancerous, though most of them are benign [1]. Lung cancer is one of the biggest malignancy cancers among all kinds of cancers in our healthy life [2, 3] and is also the most common histological type in Aden carcinoma [4]. In recent years, the number of people suffering from lung cancer increases more and more rapidly. The early stage lung cancer is shown as lung nodules, which can be discovered and treated with the assistance of computer-aided diagnostic technique in time, which will prolong the life of lung cancer patients [5, 6]. The computer-aided diagnostic scheme can detect the nodules automatically in the pulmonary CT images and decrease the miss rate [5, 7], especially with the low-dose CT (LDCT) scanning [8].

To date, many researchers all over the world are devoted to the study of the detection of attached pulmonary nodules, for example, nodule attached to vessels and the pulmonary wall. However, limitations occur in lung cancer imaging of distinguishing nodules attached to vessels from the normal blood vessels, which infiltrate the vessels surreptitiously. Using the corrosion morphology and expansion to segment the pulmonary nodules from the vessels resulted in the corrosion of the nodule thorn, which is another important index for malignant nodule valuation [9]. A weighted fuzzy C-means clustering was developed for remotely sensed image classification but requires a given number of clustering and is easy to fall into local minimum rather than the global optimal solution [10]. A method based on EM and Mean-shift or one of the two means was proposed to detect attached nodules, but there are many conditions need to be considered, and not entirely consistent with the actual situation [11, 12]. Algorithm Based on Fuzzy Integrated Active Contour Model and Hybrid Parametric Mixture Model to detect pulmonary nodules just extracts the adhesion nodules, but it did not exclude the false positives such as ends of the vessels. For the value of the pixel on the nodule which is close to that of pixel on the vessel, the gray threshold cannot work well and morphological operations cannot identify the adhesion nodules effectively [13–15]. Guo et al. developed a pulmonary nodule detection algorithm based on multiscale enhancement filtering of Hessian matrix and selecting of grads entropy, where Hessian matrix is relative to the gray scale of the pixel in the CT image, and grads entropy is also relative to gray scale of the pixel [16]. It worked well in the solitary pulmonary nodules detection, but it can only detect most suspect nodules and cannot exclude the false positives, especially the ends and the cross sections of the vessels or tracheas. Template matching method can be used to extract suspected nodules, but this will need more human intervention and prior information [17]. For solitary pulmonary nodules, regional growth can obtain good segmentation results [3]; for region growing segmentation results are part of vessel without separation and nodule. The method based on SVMS to detect the nodules, worked well, but it required a long processing time and lots of work [18–20].

Pulmonary nodules are similar to spherical objects, and the lung CT images are 2D. In order to enhance the dot-like regions and depress the line-like regions quickly and effectively, an algorithm named dot-filter was proposed by Li et al. [21]. However, when it was applied to detect pulmonary nodules, many false positives appeared, such as the ends and cross sections of the vessels and tracheas [16]. We found that the distances from the adhesion nodules center or false positives to the centerline of the vessel or tracheas were different. In this paper, starting from the relationship of their position, we combine dot-Filter and algorithm of extracting centerline, using which to identify which is the end or head of the vessel and which the circle formed by the intersection of the lines. In this way, we can separate the nodules from vessels and tracheas effectively with fewer steps.

## 2. Materials and Methods

### 2.1. Algorithms of Adhesion Pulmonary Nodules Detection

**Figure 1:**
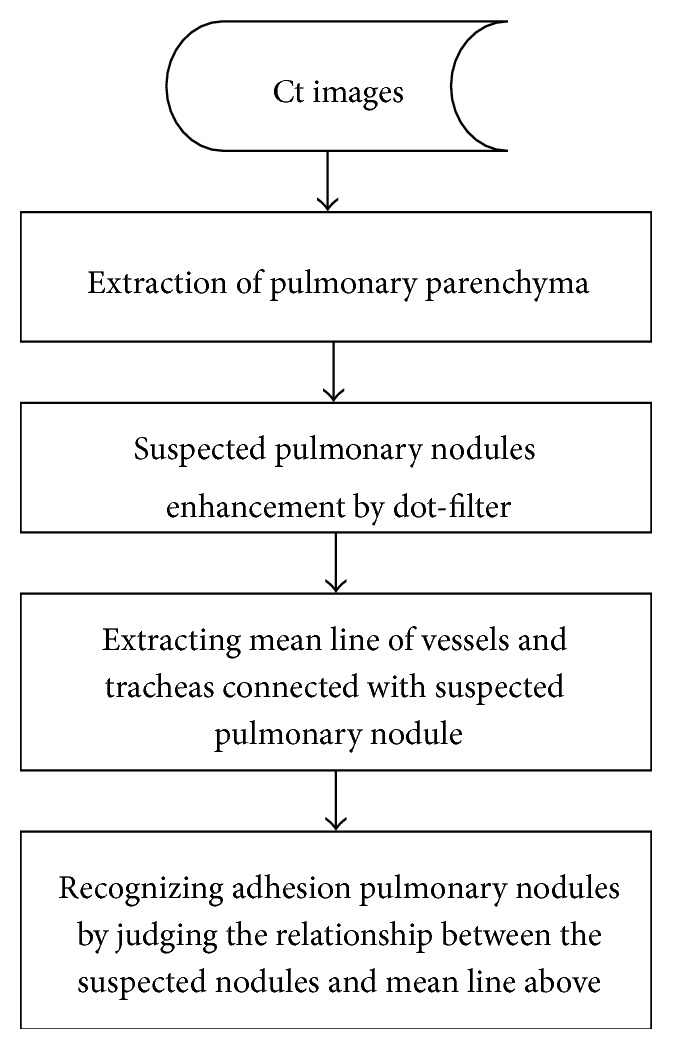
The process of the algorithms used to recognize adhesion pulmonary nodules.

The process of the algorithms used in this paper was shown in Figure 1. Firstly we removed the background noise from the initial CT images and then extract the lung parenchyma. Secondly we used the Gauss function to convolute the image and a smooth image can be obtained. After that we can use dot-filter to enhance the dot-like regions to obtain suspect nodules. At last, we used the extracting centerline algorithm to analyse the relationship of the position of the suspect nodules between the vessels and tracheas, which was used to recognize the adhesion pulmonary nodules.

### 2.2. Enhancement of Nodules by Dot-Filter

#### 2.2.1. Dot-Filter Constructed by Hessian Matrix

To a medical CT image, the enhancement filter of local structure was used extensively which is based on the shape of organization. On a 2D image, we used the dot model conforming to Gauss distribution to represent a nodule [21, 22] as well as line model; the equation is expressed as

(1) $$\begin{array}{c} d\left(x,y\right)=exp\left\{-\frac{x^{2}+y^{2}}{2\sigma^{2}}\right\},\\ l\left(x,y\right)=exp\left\{-\frac{x^{2}}{2\sigma^{2}}\right\}. \end{array}$$

**Figure 2:**
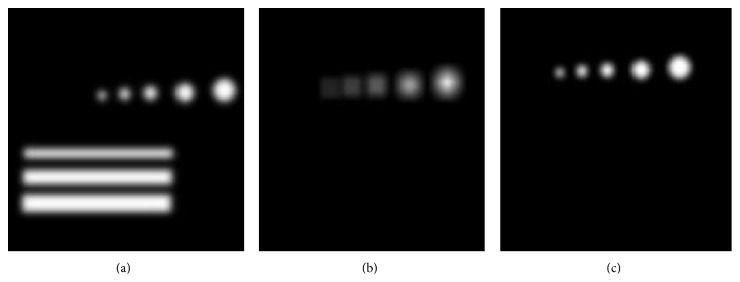
(a) simulate the model of Gaussian dot; (b) enhanced with one scale; (c) enhanced with multitude scales.

Here, *d*(*x*, *y*) denotes a dot expression expressed by a 2D Gaussian function; *σ* represents the dimension of the dot and the line. Because of the variety values of *σ*, we simulate the image of dots and lines shown in Figure 2(a).

Li et al. [21] proposed that dot-filter can be constructed by using Hessian matrix to effectively extract dot-like objects. For an original 2D image, we assume it has four second derivatives *f*_*xx*_, *f*_*xy*_, *f*_*yx*_, and *f*_*yy*_, where *f*_*xy*_ = *f*_*yx*_ and its 2D Hessian matrix is

(2) $$\begin{array}{c} H=\left[\begin{array}{cc} f_{xx} & f_{xy}\\ f_{yx} & f_{yy} \end{array}\right]. \end{array}$$

*f*(*x*, *y*) is the value of one of the pixels in the image. Suppose the Eigenvalues of *H* are *λ*_1_ and *λ*_2_ and satisfied that abs |*λ*_1_| is bigger than |*λ*_2_|. If |*λ*_1_| < |*λ*_2_|, exchange them. The |*λ*_1_| and |*λ*_2_| of the dot and line in the image satisfy the following expressions:

(3) $$\begin{array}{c} \text{dot:}\;\;\lambda_{1}=\lambda_{2}=-\frac{1}{\sigma^{2}}<0,\\ \text{line:}\;\;\lambda_{1}=-\frac{1}{\sigma^{2}}<0,\;\lambda_{2}=0. \end{array}$$

The enhanced dot-filter is expressed by the following expression [21]:

(4) wd=λ22λ1 if  λ1<0, λ2<0,wd=0  others;

In CT images, if the semidiameter of one pulmonary nodule is  *σ*_0_, the nodule will account for 49.9% of the area of the Gauss function. If it is 2*σ*_0_, it will account for 72.0% of the area of the Gauss function. And if 3*σ*_0_, it accounts for 99.0% of the area of the Gauss function. Then, to a nodule of which the semidiameter is *r*, we use one Gauss function with *σ*_0_ that equals *r*/3 to express it better [16]. For the position of the pulmonary nodules in CT images is different, the scale of the nodules is different between them. If the range of the scale of the nodules is [*r*_0_, *r*_1_], the *σ*  in Gaussian function is in [*r*_0_/3, *r*_1_/3]. In order to enhance all the goals in the range, we use different value of *σ* in Gaussian function to smooth a 2D CT image firstly; then we use the dot-filter constructed with Hessian matrix to enhance the goal area. The two steps above should be repeated *N* times with increasing scale of *σ*  from *σ*_0_ to *σ*_1_ to obtain *N* enhanced CT images. If the range becomes bigger, *N* will become bigger [16, 21]. In lung CT images, we find that the value of *N* which equals 5 is better. In the range of [*r*_0_/3, *r*_1_/3], the algorithm to obtain the *σ* can be shown as follows:

(5) σ0r03,σ1=rσ0,σ2=r2σ0, ⋮σN=rNσ0=r13;

among which the *r* equals (*r*_1_/*r*_0_)^1/(*N*−1)^. In each *N* scale, we can obtain one most effective enhancement to the appointed nodule.

The steps of extracting dot with numbers of scales of dot-filter are as follows:

1. According to the range of scale of the nodules we compute the value of *σ*.
2. For every *σ*, repeat (3)–(8).
3. Using Gaussian function convolve with 2D *f*(*x*, *y*).
4. For every pixel, repeat (5)–(7).
5. Compute *H* and |*λ*_1_|, |*λ*_2_|.
6. Compute *w*_*d*_.
7. Stop computing.
8. Select the maximum of *w*_*d*_.

In order to prove better the effect of using dot-filter with variety value of *σ* to identify the dot-like shapes, we use Figure 2(a) as input, and the output is shown as Figures 2(b) and 2(c).

Figure 2(a) is an image constructed by the expression (1) with variety scale of *σ*, and there are five dots and three lines. The scales of the *σ* among the dots are 2, 4, 6, 8, and 10 pixels.  Figure 2(b) is the image, in which the better identified dot is enhanced by one dot-filter with the scale of 10 pixels. We also found that the lines is not identified and the dots smaller than 10 pixels do not have large output.  Figure 2(c) is the image enhanced by four dot-filters, of which the dots equal to the scale of 2, 4, 6, 8, and 10 pixels all have large output, and the lines are depressed. According to Figure 2, we can prove that dot-filter can depress the line-like shapes and with variety value of *σ* it can extract all the goal areas better.

#### 2.2.2. Application of Dot-Filter Constructed

**Figure 3:**
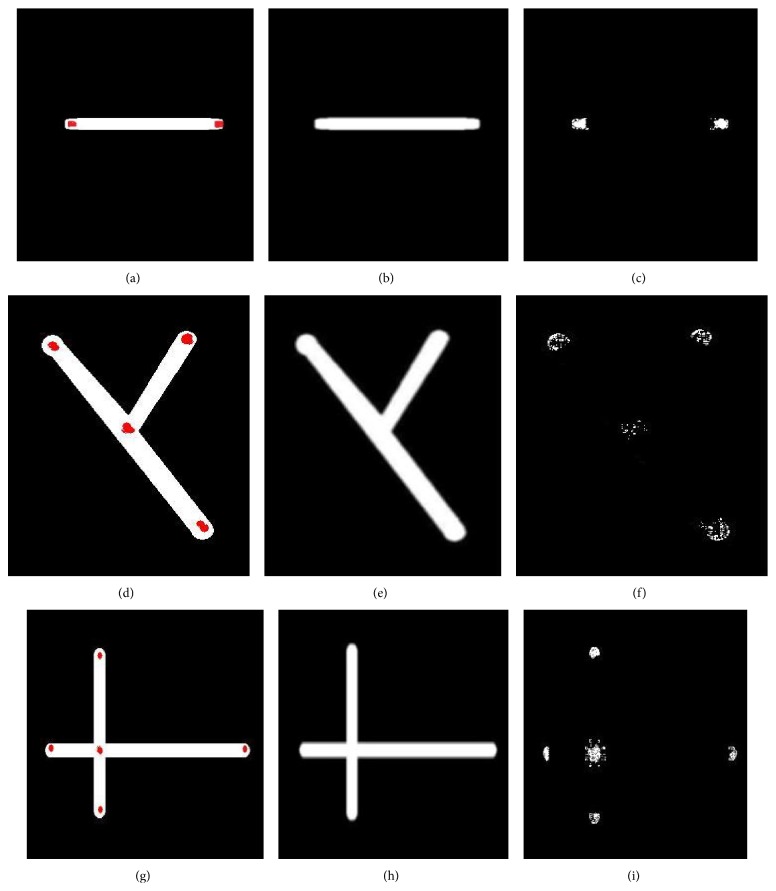
Models of vessels constructed. The regions marked by red in (a), (d), and (g) are dot-like regions, which will be enhanced by dot-filter. (a) the vessel of single line type; (b) smoothed by Gauss function; (c) enhanced by dot-filter; (d) the vessel of Y type; (e) smoothed by Gauss function; (f) enhanced by dot-filter; (g) the vessel of X type; (h) smoothed by Gauss function; (i) enhanced by dot-filter.

As depicted above, we know that dot-filter can enhance the dot-like areas effectively. However, in the lung CT images, the ends and cross sections of vessels are also of dot-like shapes, which will be enhanced by using dot-filter, leading to many more false positives appearance. In order to prove that, we construct three types' vessel models, such as single line model, Y type model, and X type model shown in Figures 3(a), 3(d), and 3(g). The regions marked by red in the images are dot-like regions, which will be enhanced by dot-filter. Figures 3(b), 3(e), and 3(h) were smoothed by Gauss function. Figures 3(c), 3(f), and 3(i) were the images enhanced by dot-filter. The enhanced areas that we marked in Figures 3(a), 3(d), and 3(g) are also called suspect nodules. Due to many suspect nodules that appeared after the enhanced process by dot-filter we need to eliminate these false positives which may lead to much more computation works.

**Figure 4:**
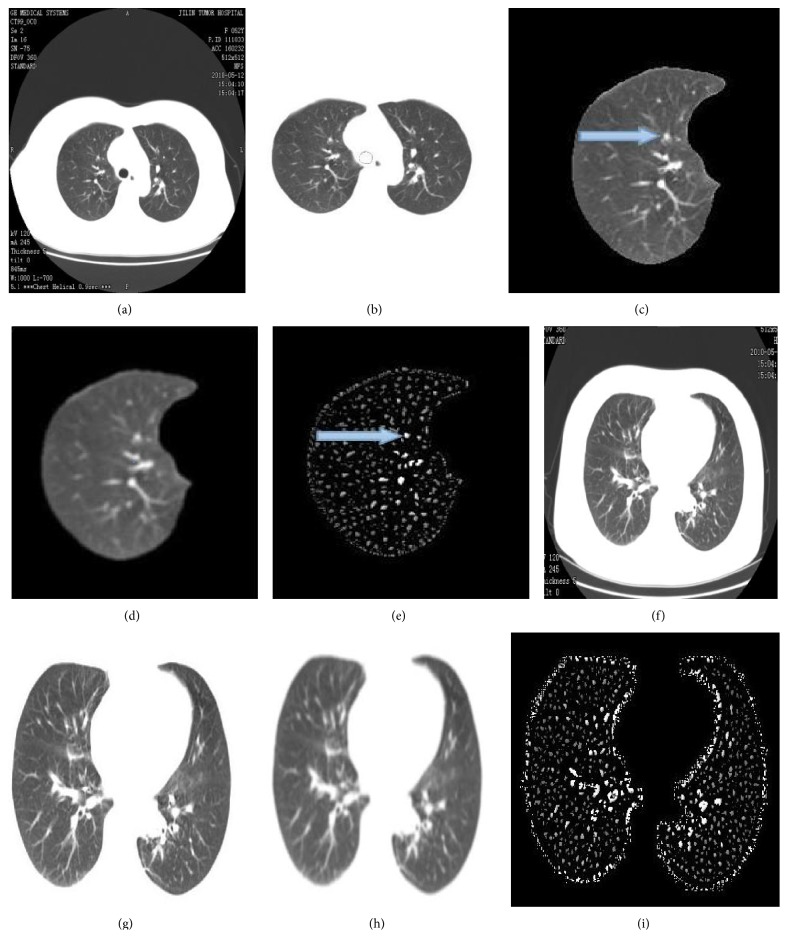
Detection of the solitary nodules in a 2D CT image enhanced by dot-Filter. (a) original lung image; (b) lung segment extraction; (c) the right lung segment; (d) lung CT image smoothed by Gauss function; (e) detection of the solitary nodules; (f) lung CT image without nodules; (g) pulmonary segment extracted; (h) smoothed by Gauss function; (i) enhanced by dot-filter.

Now we will use the Dot-Filter constructed above based on Hessian matrix to lung CT images, and the result is shown in Figure 4. Lung CT images are given by one big hospital for lung nodules detection based on dot-Filter and the algorithm of extracting centerline.

Figure 4(a) is an original pulmonary CT image.  Figure 4(b) is the pulmonary segment extracted through segmentation of digital image.  Figure 4(c) is the right pulmonary segment. In the image the blue arrow denotes a nodule identified by the doctor. From the image we can find that there are many dot-like areas such as solitary areas and dot-like areas attached to the vessels, which we take as false positives.  Figure 4(d) is smoothed by Gauss function. It is not ideal to enhance the region of interest (ROI) instantly because there is much noise in the image, so we had better firstly use Gauss function to convolute with it.  Figure 4(e) is the enhancement of the solitary nodules and other dot-like area by using dot-filter. In this image we cannot identify which is real nodule without any other assistance or algorithm, because dot-filter just enhances the dot-like areas. To effectively distinguish whether the dot-like areas are nodules or just the part of the vessels we will have to use the algorithm of centerline extracted.  Figure 4(f) is a lung CT image without nodules and Figure 4(g) is pulmonary segment extracted; Figures 4(h) and 4(i) are the image smoothed by Gauss function and enhanced by dot-filter, respectively. According to Figures 4(e) and 4(i), we obtain that dot-filter can enhance the dot-like areas effectively but leads to many false positives appearing.

### 2.3. Algorithm of Extracting Centerline

There are many algorithms used to extract the central line [23–28], such as margin of linear least square fitting legitimate, symmetric moment fitting center method, and block cancroids least squares fitting. Compared with the algorithms used in this paper, they are not stable and accurate enough and have high computational complexity. As Figure 4(e) shows, dot-filter can enhance the dot-like area significantly with more false positive increased. To overcome this shortcoming, we will combine the algorithm of extracting centerline to reduce the false positive.

#### 2.3.1. Principle

Different from the traditional algorithms of extracting central line, area skeleton can be defined by mean axle transforming (MAT). Describe an area whose profile is *b* as follows: for every pixel *p* in the *R*, we search the nearest pixel in *b*. If *p* is bigger than the nearest pixel, we named *p* centerline (skeleton) of *R*, which obeys the following constraints: (1) cannot delete the endpoint; (2) cannot destroy connectivity; and (3) cannot cause excessive corrosion of the area.

**Figure 5:**
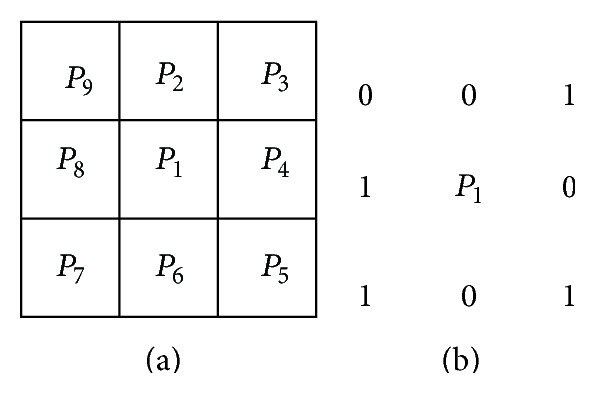
(a) The arrangement of the neighborhood pixels used in the thinning algorithm; (b) the explanation of expression (6) in conditions (a) and (b). At this time *N*(*p*_1_) = 4 and *T*(*p*_1_) = 3.

We here give the mean of a refinement of two-value algorithm region: we suppose the value of the pixel in the region is 1, and the values of the pixels on the background are 0. The value of the pixels in the edge of the region is 1 and at least there is one pixel of which the value is 0. As 8 neighborhoods shown in Figure 5(a), if it meets the following conditions (a)–(d), then (step 1) we take *p*_1_ as the pixel that will be removed as follows:

(6)  a  2≤Np1≤6, b  Tp1=1, c  p2×p4×p6=0, d  p4×p6×p8=0;

among which *N*(*p*_1_) is the number of the nonzero adjacent pixels of *p*_1_; in other words,

(7) $$\begin{array}{c} N\left(p_{1}\right)=p_{2}+p_{3}+\cdots +p_{8}+p_{9}; \end{array}$$

among which *p*_*i*_ is either 0 or 1, and *T*(*p*_1_) is the frequency conversion from 0 to 1 in *p*_2_, *p*_3_,…, *p*_8_, *p*_9_. For example, in Figure 5(b), *N*(*p*_1_) = 4 and *T*(*p*_1_) = 3.

In Step  2, (a) and (b) remain unchanged, and (c) and (d) become

(8)  c′  p2×p4×p8=0, d′  p2×p6×p8=0.

We apply step 1 to every pixel in the edge of the two-value region. If we violate (a) or (b), the value of the pixel we talk about is unchanged. Otherwise, we take it as the pixel that will be removed after we handle all the pixels of the edge. Then, we use step 2 the same way as step 1 till there is no pixel needed to be removed any more and stop the algorithm.

**Figure 6:**
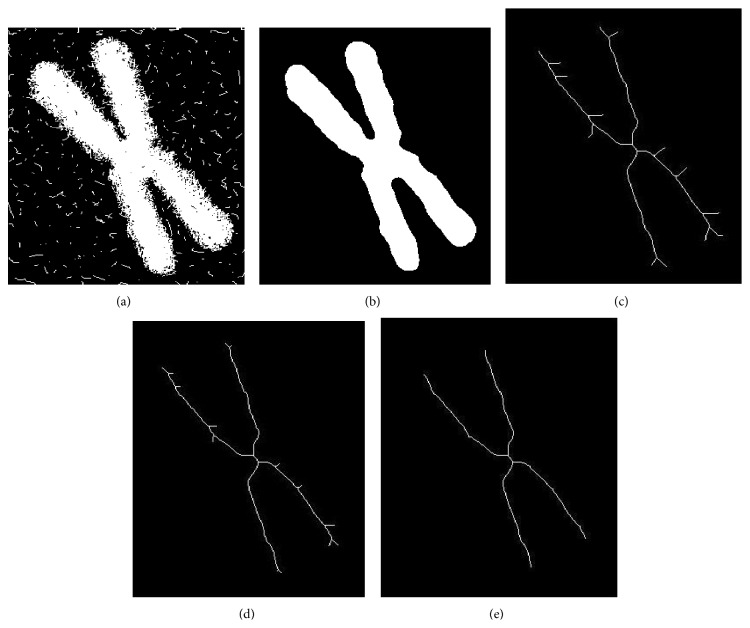
(a) chromosome image after segment; (b) image after Gaussian-filter; (c) the skeleton; (d) eight times for extinguishing the burr of the skeleton; (e) seven more times for extinguishing the burr.

Take Figure 6(a), which is processed by the mean of a refinement, for example, and the result is shown as Figures 6(b)~6(e).

Figure 6(a) is an image of a human chromosome by electron microscope magnified 30000 times and segmented using digital image processing algorithm.  Figure 6(b) is the image after Gaussian smoothing.  Figure 6(c) is the skeleton of the chromosome.  Figure 6(d) shows skeletons after applying extinguishing the burr algorithm eight times. We found that on the skeleton there is much burr but less than that in Figure 6(c). Because this algorithm is related to the threshold of the pixel, we should increase the threshold value in the algorithm.  Figure 6(e) presents seven more times for extinguishing the burr by using the algorithm.

If a line is expressed by *αx* + *βy* + *γ* = 0, we will take Δ(−*α*/*β*) and Δ(−*γ*/*β*) as the deviation [29]. If they are all very small, we will think the algorithm works better. We use other three algorithms for extracting center line to compare with the one used in this paper; the result is shown as Table 1.

According to Table 1 we found the algorithm in this paper can work effectively, though the symmetric moment fitting center method consumes the least time, but its deviation was the bigger. The algorithm used in this paper worked steadily and time consuming is not much. Combining all the factors, the algorithm used in this paper is better.

#### 2.3.2. Application

In this paper, to accomplish the experiment combining dot-filter with the method proposed above we use six steps shown as follows.

**Figure 7:**
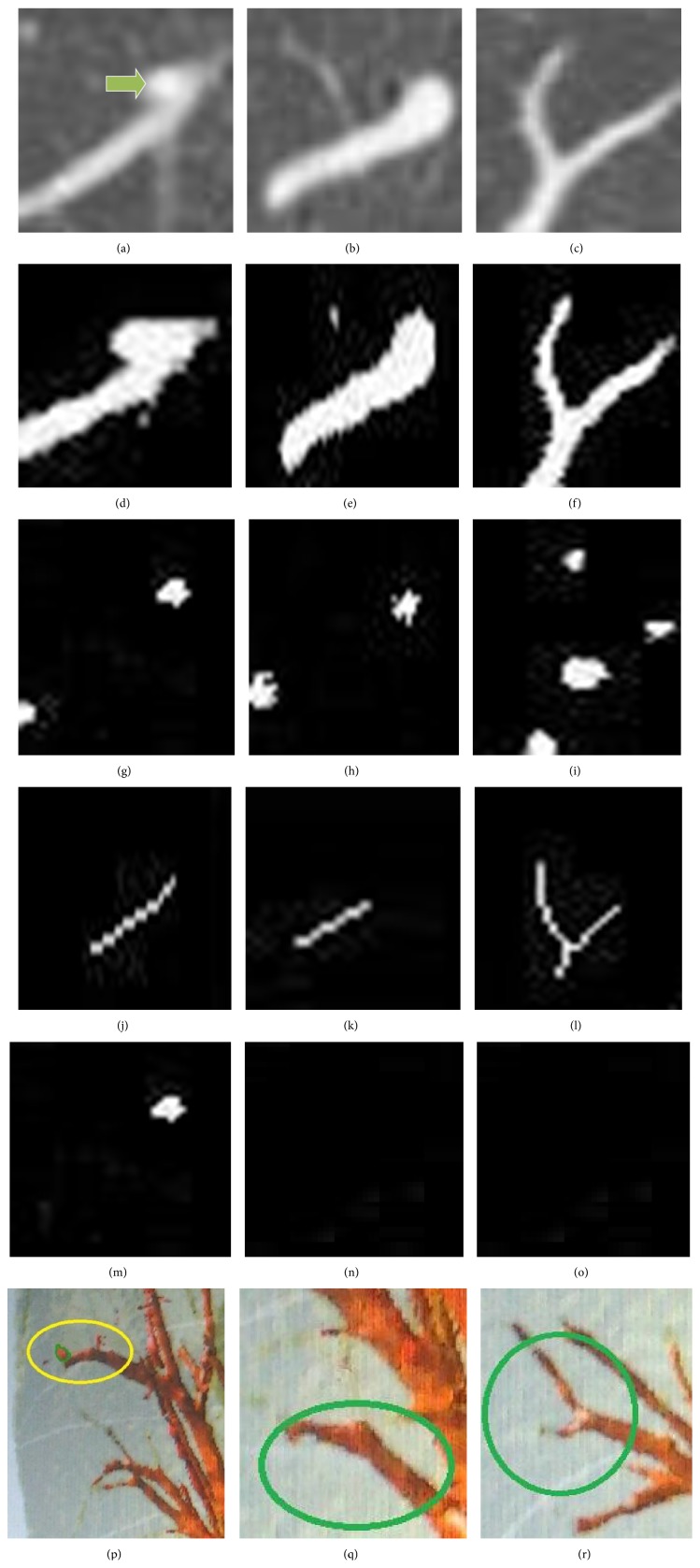
(a)~(c) original image; (d)~(f) soft tissue extraction of lung; (g)~(i) images after dot-filter; (j)~(l) images after seven times for extracting the skeleton; (m)~(o) attached nodule extraction; (p)~(r) is the three-dimension display of the objects.

1. We first selected three lung CT images after extracting the lung segment in Figures 7(a)~7(c); they were nodules attached to vessels, single vessel, and crossing vessel. There was one nodule attached to one end of the vessel noted by the doctor in Figure 7(a), shown as the arrow points to. In Figure 7(b), we can see that there is one vessel apparently and one of its ends is of dot-like shape, similar to the adhesion nodule in Figure 7(a). In Figure 7(c), the vessel is composed of two vessels and they are crossed.
2. As the value of vessels, tracheas, and nodules are bigger than the value of lung parenchyma, in order to decrease the computation, we extracted the soft tissue of the lung based on gray threshold which we defined as 130, which is obtained after many times of drawing histogram, shown in Figures 7(d)~7(f). For the low contrast nodules, we did not consider them in this paper. According to Figures 7(d)~7(f), we found that the soft tissue of the lung parenchyma was completely extracted.
3. To the tissues extracted in step (2), firstly we should extinguish the noise in the CT image. So we used Gauss filter to accomplish it. Then, we used the dot-filter constructed above to enhance the dot-like regions, in other words, we enhance the suspected nodules. The enhancement result was shown in Figures 7(g)~7(i).
4. We eliminated the tissues obtained in step  (3) from the images in step (2) and then obtained the skeleton shown in Figures 7(j)~7(l). We considered the tissues obtained in step  (3) as the false positives; we should remove them and use the algorithm of extracting centerline to extract the skeletons. But for Figure 7(f), the vessels became three parts, which was not beneficial for us to use the algorithm of extract the center line, so we firstly supplied the lack, making the vessels become one connected vessel and then extracted the skeleton.
5. Firstly we worked out the center of every suspected nodule (false positive) and then computed the value of *d*_1_, *d*_2_. *d*_1_ denotes the perpendicular distance from the center of mass of suspected nodules to the line of the skeleton near the suspected nodule obtained in step (4). *d*_2_ denotes the minimum distance from the center of mass of suspected nodules to all pixels in the skeleton obtained in step (4).
6. The diameter of the nodules is 3 mm~30 mm, so we compared the diameter with *d*_1_ obtained in (5). If *d*_1_ is smaller than 1.5 mm and *d*_2_ is smaller than 1.5 mm, the suspected nodule is treated as the intersection of the vessels or the end of the vessel. If *d*_1_ was smaller than 1.5 mm and *d*_2_ was bigger than 1.5 mm, the suspected nodule was treated as one end of the vessel. If *d*_1_ was bigger than 1.5 mm, the suspected nodule was treated as the attached nodule shown in Figure 7(m).

Figures 7(p)~7(r) is the three-dimension (3D) display of the nodule, the solitary vessel, and the crossing vessel. In Figure 7(p) the nodule with green edge is connected to the vessel in the yellow circle. Figures 7(q) and 7(r) are two kinds of different vessels in the green circle in order to be found easily. According to the three images we can easily find that the suspect nodules appeared in the process; they are actualized parts of vessels.

## 3. Results and Discussion

Table 2 depicts what are the attributes and where they come from the CT images used in this paper. 20 sets of CT images with less noise [25] were used in the experiments. They originated from LIDC database and Jida Hospital and each CT image has 512 × 512 pixels. Nodules in each CT image have been noted by doctors. The 20 true nodules and 20 false ones extracted were used to test the function of classifier. All experiments in this paper were based on the computer that consists of AMD CPU with a frequency of 2 GHz, 1.5 GB of RAM, and Windows XP operating system. Algorithm development code is developed on the platform of MATLAB.

For the CT images supported by the hospital it missed 3 adhesion nodules and missed none for the LIDC database.  Table 3 shows the missing rate and runtime of each set of CT images. Literature [9] can better extract the solitary nodules but lacks the high capacity of extracting the adhesion lung nodules because it is based on the threshold value of the pixel and it needs much justice. Literature [12] uses the algorithm which has too much computation. If we use a method with only dot-filter, there will be more false positives appearance because dot-filter can enhance the ends and the intersections of the vessels meanwhile. By using dot-filter and the algorithm of extracting the center line, we have lower missing rate and less runtime. This is because dot-filter constructed with Hessian matrix can extract the dot-like region effectively and quickly. And after extracting the center line of the vessels, we can achieve a better understanding of the relationship between the nodules and vessels. According to the relationship, it is beneficial for us to extract the nodules. There are errors of this method because scale of some nodules is very small or the value of the pixels in nodules is very small, which can lead to the miss rate increases. The method in this paper has some limitations; for example, it cannot adapt to the lower contrast nodules and the nodules attached to the lung wall. It is just applied to the nodules attached to vessels and tracheas.

## 4. Conclusion

In this paper we first use 2D Hessian matrix to construct dot-filter constructed to extract dot-like region. In order to solve the problem that the dot-filter cannot detect attached pulmonary nodules, an algorithm based on extracting centerline was used. Results of experiment indicated that the method is easy and effective while extracting attached pulmonary nodules well. In the future, we will be devoted to extracting the lung nodules contacting pulmonary wall and ground glass opacity pulmonary nodules.

## Figures and Tables

**Table 1:** The difference between four algorithms for extracting center line in deviation and time consuming.

Algorithm for center-line extracted	Δ(−α/β)	Δ(−γ/β)	*t*/s
Margin of linear least square fitting legitimate	0.142	45.437	0.042
Symmetric moment fitting center method	1.50	550.832	0.031
Block cancroids least squares fitting	0.671	332.117	0.033
Algorithm used in this paper	0.157	23.858	0.059

**Table 2:** Databases of testing the method used in this paper.

Name	LIDC database	Supported by Jida Hospital
Number of CT images	10	10
Pixel unit (volume)/mm^3^	0.6 × 0.6 × 0.6	0.6 × 0.6 × 0.6
Average number	36	70
Number of adhesion nodules	3	13
Image size/pixels	512 × 512	512 × 512
Layer thickness	1 mm	1 mm

**Table 3:** The method used in this paper compared with current two methods.

Method	Number of false positives per set	Missing rate/(%)	Runtime/(min)
Literature [9]	8.6	33.3	2.8
Literature [12]	11.2	27.5	4.2
Method with only dot-filter	34.8	18.7	1.2
Method with dot-filter and centerline extraction	5.3	18.7	1.7
